# Clinical implications of mutational analysis in gastrointestinal stromal tumours

**DOI:** 10.1038/sj.bjc.6604217

**Published:** 2008-02-05

**Authors:** A Hoeben, P Schöffski, M Debiec-Rychter

**Affiliations:** 1 Department of General Medical Oncology and Laboratory of Experimental Oncology, University Hospitals Leuven, Leuven Cancer Institute, Catholic University of Leuven, Leuven, Belgium; 2 Center for Human Genetics, Catholic University of Leuven, Leuven, Belgium

**Keywords:** gastrointestinal stromal tumours, mutational analysis, *KIT*, *PDGFRA*, targeted therapy, imatinib mesylate

## Abstract

The management of localised and advanced gastrointestinal stromal tumours (GISTs) in terms of histological diagnosis, surgery, imaging, medical treatment and molecular biology has rapidly changed since introduction of imatinib mesylate for molecularly targeted therapy in 2000. In this minireview, we briefly summarise and discuss the current data relevant to the increasing role of molecular characterisation of GISTs in the diagnosis, risk assessment and effective targeted therapy.

Gastrointestinal stromal tumours (GISTs), the most common mesenchymal tumours of the gastrointestinal tract, hypothetically evolve from a progenitor related to the interstitial cells of Cajal. Population-based studies reported GIST annual incidence rates ranging from 6.5 to 14.5 per million (Joensuu, 2006). The most common sites of origin for GIST are stomach (39–70%) and small intestine (31–45%), but GISTs may arise anywhere along the gastrointestinal tract or within the abdomen as extragastrointestinal tumours (Demetri *et al*, 2007). Frequent clinical symptoms include bloating, gastrointestinal tract bleeding, fatigue and anaemia. Gastrointestinal stromal tumours are highly resistant to traditional chemotherapeutic agents. The usefulness of radiotherapy is also very limited in the therapeutic setting mostly due to toxicity to the surrounding structures (Joensuu *et al*, 2002). Surgery has been the basis of treatment for GISTs. The 5-year survival rate is only 54% after complete resection of a localised primary GIST (Dematteo *et al*, 2002). The median survival for patients with high risk and overtly malignant tumours is <2 years and <4 years, respectively. Independent prognostic factors that predict the tumour-free survival are tumour size and proliferative index (Nilssen *et al*, 2005). The common metastatic sites for GIST include the liver and omentum. Due to recognition of the disease as a separate entity, the diagnosis of GIST has dramatically enhanced since 1998, and survival has greatly improved since introduction of imatinib mesylate in 2000.

## PATHOGENESIS, DIFFERENTIAL DIAGNOSIS AND RISK ASSESSMENT

Identification of *KIT*-activating mutations as a key factor in the pathogenesis of GIST has substantially altered the diagnosis and treatment of GIST (Hirota *et al*, 1998; Corless *et al*, 2004; Miettinen and Lasota, 2006). Oncogenic *KIT* mutations are detectable in 75–85% of all GISTs, even in small, incidentally discovered lesions. These mutations most frequently involve the intracellular juxtamembrane domain of the receptor encoded by exon 11 (57–70%), followed by the extracellular domain encoded by exon 9 (5–18%). The *KIT* exon 11 mutations are quite heterogeneous, encompassing mainly in-frame deletions of variable sizes, basic amino acid substitutions, or more complex deletions–insertions. The *KIT* exon 9 mutations represent mainly in-frame tandem duplication, AY502-503dup. *KIT* mutations in the split kinase domains I and II (encoded by exon 13 and exon 17) appear to be rather uncommon, accounting for 0.6–1.4% of all mutations. Alternatively, a subset of GISTs (5–10%) carries constitutively activating mutations in the gene encoding platelet-derived growth factor receptor *α* (PDGFRA), a receptor tyrosine kinase homologous to KIT (Heinrich *et al*, 2003b). The mutations reported within *PDGFRA* involve exon 12, 14 and 18, being homologous to *KIT* exon 11, 13 and 17, respectively. In >90% of cases, *PDGFRA* mutations target codons 842–849 (exon 18), with the D842V substitution being most common. Gastrointestinal stromal tumours that harbour different *KIT* or *PDGFRA* mutations have different molecular signatures at the level of gene expression, which further contributes to the complexity of GIST biology and variable response to treatment (Antonescu *et al*, 2004).

Approximately 10–15% of GISTs that arise in adults lack detectable mutations of *KIT* or *PDGFRA* (referred to as wild-type GISTs). Notably, wild-type genotype is a characteristic feature of the vast majority of GISTs, which are diagnosed in children and adolescents and GISTs associated with familial syndromes such as neurofibromatosis, Carney–Stratakis syndrome or the Carney Triad (Corless *et al*, 2004; Miettinen and Lasota, 2006).

Histopathologically, GISTs are a heterogeneous group of tumours, featuring spindle cell, epithelioid or mixed type morphology, and showing a wide clinical spectrum from benign to frankly malignant sarcomas (Fletcher *et al*, 2002; Miettinen and Lasota, 2006). Gastrointestinal stromal tumours typically show expression of CD117/KIT (95%) and frequently CD34 (70%) antigens by immunostaining, yet a small fraction of GISTs lack both diagnostic markers (Hornick and Fletcher, 2007). KIT-negative GISTs have a predilection for the stomach and omentum and are most commonly epithelioid or mixed cell in type. Most KIT-negative GISTs harbour *PDGFRA* mutations (80%) (Heinrich *et al*, 2003b; Corless *et al*, 2005); the others contain *KIT* mutations. The diagnosis of KIT-negative GIST can be problematic. Yet, some of the mutations detected in KIT-negative GISTs (including some *PDGFRA* mutations) are known to be imatinib sensitive; therefore, the precise diagnosis is of utmost importance and lack of immunoreactivity for KIT in a GIST should not be used as justification to deny patients therapy with imatinib. In this context, mutational analysis is mandatory to confirm the diagnosis of GIST.

The most important prognostic features of GIST in regard to the malignant potential and prognosis are tumour size (diameter of the largest mass) and mitotic index per high-powered field (Fletcher *et al*, 2002). In addition, Miettinen and Lasota (2006) confirmed the results of earlier studies indicating that the anatomic location affects the risk of disease recurrence, that is, the gastric tumours showing better prognosis than small intestinal tumours. Yet, even low risk tumours still may have a malignant potential and the prediction of the biological behaviour of GISTs is difficult based on pathomorphological criteria alone. Recent studies indicate that the type of tumour mutation may be an additional prognostic risk factor for GISTs. Thus, the deletions in *KIT* exon 11, particularly those involving codons 557–558, are associated with high metastatic risk and poor prognosis, whereas *KIT* exon 11 missense mutations are correlated with longer progression-free survival (PFS) and better overall survival (Singer *et al*, 2002; Martin *et al*, 2005; Andersson *et al*, 2006; Miettinen and Lasota, 2006; Steigen *et al*, 2007). In addition, duplications in the distal part of *KIT* exon 11 lead to a less aggressive phenotype. Conversely, the presence of homo-/hemizygous *KIT* exon 11 mutations is associated with an increased risk for metastasis and with an adverse clinical course (Lasota *et al*, 2007). Gastrointestinal stromal tumours that are defined by a *PDGFRA* mutations occur almost exclusively in the gastric location, show epithelioid morphology and usually have a low mitotic count. In most cases, PDGFRA-mutated GISTs are characterised by a low malignant potential (Lasota *et al*, 2004, 2006). Therefore, detection of a *PDGFRA* mutation in gastric GISTs may represent an additional prognostic marker of more benign tumour behaviour.

## TREATMENT – RESPONSE ACCORDING TO MOLECULAR SUBTYPE

The *KIT* or *PDGFRA* mutations in GISTs differ in type and affect different receptor domains (Corless *et al*, 2004). It is well known that sensitivity to imatinib depends on the location of the *KIT*/*PDGFRA* gene mutation. Imatinib effectively inhibits KIT with the juxtamembrane-type mutation, but fails to inhibit KIT with certain activation loop (exon 17) mutations, such as D816V in mastocytosis (Ma *et al*, 2002; Heinrich *et al*, 2003a, 2003b, 2006a). Similarly, imatinib efficiently inhibits the juxtamembrane-type PDGFRA mutations, while some PDGFRA tyrosine kinase domain II mutations (mainly point mutations involving codon 842, for example, D842V) confer primary resistance to imatinib (Debiec-Rychter *et al*, 2005; Heinrich *et al*, 2006a). The phenomenon can be explained by the concept that some tyrosine kinase domain II-type mutations induce stabilisation of the activation loop in an active conformation and/or structural alteration at the imatinib-binding site of KIT, resulting in a decreased affinity for imatinib. Moreover, experimental *in vitro* studies have shown that different amino-acid substitutions in *KIT* juxtamembrane domain, even at the same codon, might also cause different structural alteration and lead to different imatinib sensitivities (Nakagomi and Hirota, 2007). These findings should be an important factor to consider while determining the eligibility for adjuvant or neoadjuvant imatinib therapies, as patients with tumours harbouring mutations primarily resistant to imatinib will not be benefited from the treatment. Recently, neoadjuvant approaches to downsize GISTs prior to surgical resection and the adjuvant treatment of intermediate- and high-risk tumours after complete or almost complete surgical resection are evaluated in a few international clinical trials; both approaches may possibly become routine clinical practice in the future.

Even more importantly, data from randomised, multicenter North American and EORTC-Australian phase II/III clinical trials for patients with unresectable or metastatic GISTs provided clear evidence that tumour mutational status is associated with outcome of imatinib therapy (Heinrich *et al*, 2003a, 2003b; Debiec-Rychter *et al*, 2006). Patients with GISTs harbouring *KIT* exon 11 mutations have higher response rates and longer PFS than those whose tumours carry *KIT* exon 9 mutations or with no detectable mutations. Notably, the EORTC-Australian 62005 study demonstrated that patients with *KIT* exon 9 mutations, but not those with other tumour genotype, had significantly longer PFS when treated with imatinib 800 mg day^−1^ compared with 400 mg day^−1^ (Debiec-Rychter *et al*, 2006). The significant PFS advantage observed with the 800 mg day^−1^ dose in patients with *KIT* exon 9 mutations in the EORTC-Australian study was not confirmed in the North American S0033 study. However, it remained significant (*P*=0.017) when the pooled data set was examined further in a meta-analysis of 1640 patients who had been followed for a median of 45 months (Van Glabbeke *et al*, 2007).

## PREDICTIVE VALUE OF MUTATIONAL ANALYSIS FOR IMATINIB-RESISTANT GISTS

Resistance of GIST tumours to imatinib treatment is emerging as a clinical challenge.

Early resistance has been reported in 10–20% of cases (Demetri *et al*, 2007). However, the vast majority of responding patients will eventually develop secondary tumour progression. Development of imatinib resistance can follow several patterns, including progression at the primary tumour site or the development of new metastatic lesions. Available data suggest that many cases of focal progression during imatinib therapy result from clonal evolution (Antonescu *et al*, 2005; Debiec-Rychter *et al*, 2005; Heinrich *et al*, 2006a; Wardelmann *et al*, 2006). Depending on the series of patients recently published, the PFS under imatinib treatment ranges between 7 and 53 months.

Late imatinib resistance is most commonly associated with acquisition of secondary KIT mutations in the split kinase domains I and II (exon 13, 14 or exon 17). Some of these mutations alter specifically the configuration of the ATP-binding kinase pocket (V654A and T670I), inhibiting imatinib binding. Others stabilise the active conformation of the receptor, which also prevents imatinib binding (D820Y and N822K). Secondary *PDGFRA* mutations have also been described (Debiec-Rychter *et al*, 2005; Heinrich *et al*, 2006a). Importantly, several different types of mutations may occur independently indicating polyclonal resistance (Heinrich *et al*, 2006a; Wardelmann *et al*, 2006). It has been shown that GISTs with an underlying primary *KIT* mutation in exon 11, known to be the subgroup with better response rates than other mutational subtypes, reveal more frequently secondary mutations compared to tumours with an underlying exon 9 mutation (Antonescu *et al*, 2005; Debiec-Rychter *et al*, 2005; Wardelmann *et al*, 2006). This observation suggests that the development of a secondary *KIT* mutation is an important escape mechanism for tumour cells of which KIT-dependent proliferation is chronically inhibited by imatinib.

Another putative mechanism of imatinib mesylate resistance is *KIT* gene amplification (Debiec-Rychter *et al*, 2005). In addition, it is proposed that other oncogenes or tumour suppressor genes may have become important in sustaining the tumorigenic potential, rendering the tumour independent from KIT signalling and thus, making it insensitive to imatinib treatment (Heinrich *et al*, 2006a). Identifying altered expression of genes, known to be important in tumorigenesis, is an important research area that could lead to validation of interesting targets for specific molecular therapies.

Since secondary resistance of GIST patients to imatinib is a growing clinical problem, multiple novel inhibitors are in development to interfere with kinase signalling using alternative KIT and/or PDGFRs inhibitors or by targeting critical downstream-signalling proteins to regain disease control after failure of imatinib (von Mehren, 2006).

Sunitinib mesylate (SU011248, Sutent®, Pfizer) is approved for imatinib-refractory GIST. Sunitinib is an oral tyrosine kinase inhibitor that targets multiple kinases, including the vascular endothelial growth factor receptors (VEGFR-1, VEGFR-2 and VEGFR-3), PDGFRs, KIT, FLT3 and the receptor encoded by the *RET* proto-oncogene. It has both antiangiogenic and antiproliferative activities (Goodman *et al*, 2007). A phase III double-blind trial comparing sunitinib with placebo was conducted in 312 patients with GIST who had documented failure or intolerance of imatinib (Demetri *et al*, 2006). Only 5% of the patients who had imatinib-resistant GIST showed objective response, but 58% had disease stabilisation. Importantly, patients with imatinib-resistant GISTs with *KIT* exon 9 mutations may benefit more from sunitinib than those with an exon 11 mutation. In the patient group reported by Heinrich *et al* (2006b), 37% of the imatinib-resistant GISTs with a primary exon 9 mutation responded to sunitinib, as compared with 5% of cases with a primary exon 11 mutation. PFS and overall survival were also longer for patients with GIST with either a primary *KIT* exon 9 mutation or with no detectable *KIT*/*PDGFRA* mutation, compared with tumours with a *KIT* exon 11 mutation. The confounding factor might be a higher incidence of secondary *KIT* mutations in exon 11-mutant tumours, which may reflect longer duration of imatinib therapy and therefore increase chances for selection of mutations producing imatinib resistance in these tumours. Sunitinib shows lower efficacy for secondary *KIT* mutations in exons 17 and 18 in comparison with secondary mutations in exons 13 and 14 (Prenen *et al*, 2006; Heinrich *et al*, 2006a, 2006b). Given that GIST progression is a polyclonal event and a variety of secondary, imatinib-resistant *KIT* mutations may be present in parallel (Wardelmann *et al*, 2006), sunitinib may not be efficient to inhibit the proliferation of the different tumour clones present. As it will be impossible to identify all emerging resistant tumour clones, *KIT* sequencing has only a limited role for predicting clinical benefit of sunitinib, and the same holds true for the other second-line TK inhibitors.

Nilotinib (AMN107, Novartis) is an oral tyrosine kinase inhibitor that inhibits downstream signalling of BCR-ABL, KIT and PDGFRs. Preclinical studies have shown better efficacy of Nilotinib against certain *KIT* and *PDGFRA* mutations in comparison with imatinib (Weisberg *et al*, 2006; Guo *et al*, 2007).

Dasatinib (BMS-354825, Bristol-Myers Squibb) is a dual SRC/ABL inhibitor. This drug binds to the active conformation of KIT to which imatinib cannot bind. In this perspective, dasatinib could be a valid therapy for GIST patients expressing a secondary *KIT* mutation that stabilises the receptor in its active conformation form (Schittenhelm *et al*, 2006).

Sorafenib (BAY 43-9006, Nexavar®, Bayer) is a novel biaryl urea compound that was initially developed as a specific inhibitor of serine–threonine kinase RAF. In addition, sorafenib inhibits multiple receptor tyrosine kinases, including VEGFR-2, VEGFR-3, PDGFRs and KIT. Preclinical data show that sorafenib is more efficient, compared to nilotinib and dasatinib, to inhibit the downstream signalling of KIT receptors containing imatinib-resistant secondary mutations (Guo *et al*, 2007).

Heat-shock protein 90 inhibitors prevent heat-shock protein 90 from stabilising client proteins, like KIT. The targeted proteins are then increasingly directed towards the proteasome for degradation. Preclinical data show that these compounds are able to decrease downstream signalling from the receptor that contains primary and secondary imatinib-resistant mutations by increasing KIT degradation (Bauer *et al*, 2006). Clinical trials in imatinib-resistant GIST patients are ongoing.

Additional molecular characterisation of GISTs becomes increasingly important to identify which of the immense variety of newly developed tyrosine kinase inhibitors might be successful for the treatment of imatinib resistant GISTs. Moreover it has become clear that identifying additional genomic alterations and establishing protein and gene expression profiles in GIST might ultimately help identify new markers of tumour behaviour, prognosis and drug response.

## MUTATIONAL ANALYSIS OF GISTS IN THE MANAGEMENT OF THE DISEASE

The molecular characterisation of GISTs has become an essential part of the routine management of this disease.

At first diagnosis of resectable disease, mutational analysis mainly serves academic purposes, as the prognostic assessment of individual cases at present is based on solid clinical and morphological variables. However, according to recent NCCN clinical practice guidelines, GIST mutational analysis is strongly recommended for the primary intermediate or high-risk tumours (Demetri *et al*, 2007). Additionally, in selected cases with atypical histopathological or clinical features, molecular studies are required to help make a clear diagnosis. Molecular techniques are also essential to establish the diagnosis of emerging subtypes of GIST, and to differentiate them from common GIST or other mesenchymal malignancies.

At first diagnosis of disseminated, unresectable disease, mutational analysis should be considered a standard of care, as various studies have clearly shown that the type of mutation of GISTs clearly correlates with key clinical outcome parameters, such as response rate and PFS. While the results of molecular studies might not have an immediate impact on the choice of drug or daily dose, due to non-availability of approved treatment alternatives to imatinib 400 mg in most health care systems, patients with ‘poor response genotype’ might be considered for more intense follow-up, with higher frequency of early imaging assessments, including computer tomography and/or FDG-PET. This helps in predicting and identifying the treatment failure and to switch to a more appropriate treatment as early as possible, and could even reduce overall treatment costs.

In patients progressing during treatment with standard doses of imatinib who potentially qualify for dose escalation, and in patients failing the highest available doses of the imatinib who are potential candidates for treatment with sunitinib, mutational analysis of progressing lesions is of academic purposes. The comparison between the initial genotype with the mutational profile of refractory lesions might contribute to the further understanding of the natural biology and evolution of GIST and other solid tumours.

Patients failing all conventional systemic treatment options at present qualify for clinical trials involving new targeted agents or innovative drug combinations. In such an experimental setting, it is absolutely crucial to gain as much information about the treated tumour as possible, enabling the researcher to interpret the outcome of the study in the most appropriate way. In this situation molecular studies are generally regarded essential. This can involve analysis of historical tumour material, fresh biopsies of progressing lesions prior to study entry or even sequential biopsies during the conduct of the trial. The molecular profile of the tumour can be used at study entry to enrich the patient population for better outcome, which can help to establish the real value of a new drug or combination. The same principle holds true for trials focusing on preoperative (neoadjuvant) or postoperative (adjuvant or additive) use of targeted agents in this malignancy, where molecular studies can have major impact on trial outcome and interpretation of the study data.

A major limitation though is the global nonavailability of cross-validated methodology used for mutational analysis in GIST. It is an absolute requirement to overcome this issue by sharing biological material, co-analysing samples, comparing the results and establish common standards between reference laboratories. Such projects are considered within the framework of various academic and commercial groups, but at present without visible outcome.

In conclusion, the type of *KIT* or *PDGFRA* mutation appears to influence tumour development and response to current therapies and, therefore, might have profound power in assessing the prognosis of the disease. Currently, *KIT/PDGFRA* mutational analysis is mandatory for KIT-immunonegative GISTs and strongly recommended for the primary intermediate or high-risk tumours. With newly developed small molecule inhibitors or alternative drug strategies, mutational analysis may become indispensable for rationale and effective GIST treatment in the future.
